# Some Hospital Finance in 1908

**Published:** 1909-01-02

**Authors:** 


### HOSPITAL ADMINISTRATION.

### CONSTRUCTION AND ECONOMICS.

### SOME HOSPITAL FINANCE IN 1908.

THE proceedings at the annual meeting of the Presidents of the League of Mercy, which we reported last week, were full of interest. The League of Mercy was established in 1899 to afford an absolute guarantee to small subscribers that all the money they may give will go without deduction into the coffers of the voluntary hospitals. It was held by many at the time that it was impossible to organise a system whereby annual subscriptions of one shilling could be collected and a large revenue raised, but the fallacy of this contention has been demonstrated by the remarkable success and continuous growth of the sums raised each year by the League of Mercy. Sir William Collins showed that the League has succeeded in attracting the small subscribers, and gave as an example the experience of one Vice President of a poor district who wrote to say that nearly £20 of the sum included in his cheque consisted of the contributions of 4,000 poor people in penny subscriptions. The experience of the League throughout the Metropolis and in the country districts has been that poor people will give readily, according to their means, now that they have come to understand through the work of the League of Mercy that their help is really needed, and that all their contributions go directly, without any deduction, to the hospitals for which they are intended. The Treasurer of the League had the gratification of reporting that the total amount raised in 1908 by the League of Mercy would exceed £22,000 and so constitute a record. Of this sum £20,516 has been distributed in grants among metropolitan and country hospitals. Altogether during the ten years of its existence the League of Mercy has raised and distributed £119,530.

It is remarkable to notice that in the *Standard* of the 18th ult. the Hon. Sydney Holland and Sir Edmund Hay Currie set themselves to show that during the year 1908 there has been a check in the flow of charity to the hospitals. We venture to say that, though there may have been a falling-off in the sum received through the ordinary channels by certain hospitals, including the London Hospital, and by the Hospital Sunday Fund, the actual volume of charity has, in fact, not diminished. Further than this, as the League of Mercy Report shows, there never was a time, probably, when so many people gave deliberately to the support of voluntary hospitals, each year, as to-day. Before the establishment of the King's Fund and the League of Mercy the tendency was to narrow too much the constituency from which voluntary gifts were invited. The influence and work of these two great organisations have tended to increase considerably the number of givers to the hospitals, and through their instrumentality, mainly, there has never been a time, in the history of British charity, when the voluntary system of hospital support rested on so sure a foundation as it now does.

Where individual charities have suffered in revenue the cause is usually traceable to a want of elasticity in the system pursued in the raising of funds. In our view the London Hospital makes a great mistake in continuing the old system of a five years' maintenance fund. This method is really obsolete, as we ventured to show in our issue of February 8, 1908. The London Hospital requires an increased revenue from voluntary sources of between £25,000 and £30,000 a year. That money could be and ought to be raised by a financial organisation continuously at work, in place of the enervating effort to raise a large fund every fifth year. In the same way the Hospital Sunday Fund should set itself to make the public understand that the Herring bequest ought only to be regarded as a bonus given by a deceased benefactor, who was interested in that Fund, in order to encourage the living to do their part adequately each year, by raising the total sum available for distribution by the Sunday Fund in London to at least £100,000.

Mr. George Alexander, the eminent actor, whose public spirit in devoting a measure of his time to public affairs is to be highly commended, made a most practical speech at the League of Mercy meeting, full of helpful hints to Hospital Committees and Managers. His theme was that benefit performances should never be sanctioned on the part of the management of any charity until the Committee knew that such a performance was really legitimate, and that the money raised would be paid to the Charity. The experience of managers of the London theatres is that most of the applications made require to be very carefully sifted, for there is nothing that is so much abused as the charity performance. It is essential first of all to ascertain what is the real underlying motive for each application. Is it to please or benefit some individual, or does it rest solely upon a desire to benefit the particular hospital or charity? Mr. Alexander has found it necessary to make a rule, so far as his own theatre is concerned—and he commends this rule to the adoption of all managers of hospitals and charities generally—that those who are getting up a performance should guarantee the expenses of the entertainment in advance, so that the money paid at the doors shall go directly and without deduction to the Charity for which it is intended. We have heard of one such performance for the benefit of a hospital where upwards of £1,100 was received, of which the promoter took 10 per cent., though the actual sum handed to the Charity was under £100. Mr. Alexander quoted an instance where the money received amounted to £562, but the Charity only obtained £90. In another case where the management was *bona fide*, and Mr. Alexander's system was followed, the tickets realised £1,400 and the Charity obtained £1,380 of that sum, the expenses amounting to £20 only.

We hope the Committee of every Hospital and Charity, as well as the Presidents of every branch of the League of Mercy, will take this warning of Mr. Alexander's to heart, for otherwise the Hospitals must suffer, not only directly by the loss of money which they ought to receive, but indirectly by diminished revenues from people, who, if they did not take tickets, might subscribe regularly to the funds of the hospital. It must never be forgotten in regard to these benefit performances, as Mr. Alexander insisted, that there are many people who take tickets for a performance on behalf of this or that hospital, who feel that in doing so they have done everything that can be expected of them, so far as that particular hospital is concerned. Our experience is that in the majority of cases Hospitals and Charities would be infinitely better off without these benefit performances, and no Charity should be identified with any such undertaking until its managers have secured the guarantee upon which Mr. Alexander insisted with so much force and judgment. All interested in charity are indebted to Mr. Alexander for his public-spirited action in this matter.

